# Long-Term DL-3-*n*-Butylphthalide Treatment Alleviates Cognitive Impairment Correlate With Improving Synaptic Plasticity in SAMP8 Mice

**DOI:** 10.3389/fnagi.2018.00200

**Published:** 2018-07-05

**Authors:** Chaonan Lv, Qinying Ma, Bing Han, Jing Li, Yuan Geng, Xiaoman Zhang, Mingwei Wang

**Affiliations:** ^1^Department of Neurology, the First Hospital of Hebei Medical University, Shijiazhuang, China; ^2^Brain Aging and Cognitive Neuroscience Key Laboratory of Hebei Province, Shijiazhuang, China

**Keywords:** DL-3-*n*-butylphthalide, synaptic plasticity, Alzheimer’s disease, SAMP8, BDNF

## Abstract

Alzheimer’s disease (AD) is the most prevalent form of dementia worldwide. AD is characterized by mild cognitive impairment at onset, irreversibly progressing with age to severe neurodegeneration and cognitive deficits in the late stages. Unfortunately, no effective treatments exist to prevent or delay the cognitive symptoms of AD. Studies have shown that DL-3-*n*-butylphthalide (DL-NBP) alleviates cognitive impairment induced by amyloid-β in mice by reducing oxidative stress, inhibiting apoptosis, and decreasing tau phosphorylation. In this study, we examined the effects of DL-NBP administration on cognitive function in the senescence-accelerated mouse prone 8 (SAMP8) model of age-related dementia. DL-NBP treatment for 3 months alleviated cognitive impairment in SAMP8 mice as assessed by performance in the Morris water maze test. Moreover, DL-NBP significantly increased the expression of synaptophysin and postsynaptic density protein 95 in the hippocampus of SAMP8 mice, indicative of a protective effect on hippocampal structural synaptic plasticity. In addition, brain-derived neurotrophic factor/tropomyosin receptor kinase B signaling, previously shown to promote synaptic plasticity, was significantly enhanced by the DL-NBP administration. Our findings suggest that DL-NBP is a potential drug candidate for the treatment of cognitive impairment in AD and may serve as the foundation for further research into the development of AD drugs.

## Introduction

Alzheimer’s disease (AD) is a destructive and burdensome age-dependent neurodegenerative disorder characterized by mild cognitive impairment at onset and irreversible neurodegeneration and dementia in the late stages (Gainotti et al., 2014). The etiology of AD remains unclear, and no effective means of prevention or treatment exist.

The senescence-accelerated mouse (SAM), developed by Professor Toshio Takeda at Kyoto University, is an accelerated-aging model based on aging instead of gene mutation (Takeda et al., 1997). The SAM consists of the senescence-accelerated mouse prone (SAMP) and senescence-accelerated mouse resistant (SAMR) strains. SAMR1 mice age normally, while the SAMP8, a substrain of the SAMP, shows early-onset irreversible aging following a normal development process. In particular, SAMP8 animals exhibit rapid deterioration of memory and learning ability (Hosokawa et al., 1984). As a result of accelerated aging, SAMP8 mice display a number of characteristics that occur early in the pathogenesis of AD, such as loss of neurons and dendritic spines, cholinergic defects, increased oxidative stress, and tau phosphorylation (Akiguchi et al., 2017). Because 95% of AD cases are sporadic, have multiple causes, and occur in patients aged ≥65 years, the SAMP8 strain provides a useful model of AD and other age-related human diseases (Pallas et al., 2008).

Synapse loss occurs at the early stage of AD, worsens progressively, and correlates closely with the cognitive decline (DeKosky et al., 1996; Selkoe, 2002; Coleman and Yao, 2003; Duits et al., 2018). Synaptic plasticity describes changing strength of the connection between two neurons. As a functional term, it refers to alterations in synaptic efficiency; however, physiological changes in transmission strength are often accompanied by structural changes in synapses. Since memory is thought to be stored in the brain synapses, synaptic plasticity is considered the biological basis of learning and memory. Age-related cognitive decline is associated with a gradual decrease in the structural and functional plasticity of the hippocampus (Bisaz et al., 2013). Synaptophysin (SYN) and postsynaptic density protein 95 (PSD-95) are pre- and postsynaptic terminal markers, respectively. Brain-derived neurotrophic factor (BDNF) can effectively regulate synaptic plasticity, learning, and memory, in addition to its classical trophic activities (Lohof et al., 1993; Figurov et al., 1996). The expression and activity of synaptic proteins may be regulated via BDNF binding to tropomyosin receptor kinase B (TrkB), located at the presynaptic and postsynaptic sites (Drake et al., 1999; Pozzo-Miller et al., 1999). Several studies have shown that BDNF/TrkB signaling is attenuated in the early stages of AD. Thus, the expression of SYN and PSD95 in the hippocampus may be increased by enhanced BDNF/TrkB signaling (Chen et al., 2017).

Developing new drugs to prevent the cognitive decline in AD is challenging: the process is lengthy, expensive, and has a high failure rate (Liu et al., 2014; Wisniewski and Goni, 2015). Repurposing older drugs for new indications might provide a lower-risk alternative (Ashburn and Thor, 2004). DL-3-*n*-butylphthalide (DL-NBP) was initially approved by the State Food and Drug Administration of China (SFDA) for stroke therapy in 2005. DL-NBP is derived from L-3-*n*-butylphthalide, which was initially isolated as a pure component from the seeds of *Apium graveolens* in 1978 by researchers at the Institute of Medicine of the Chinese Academy of Medical Sciences. Data collected in stroke animal models over the past few years have revealed that DL-NBP can increase the brain microcirculation, inhibit inflammation, and reduce oxidative damage, mitochondrial dysfunction, and apoptosis (Xiong et al., 2012; Xu et al., 2012; Wang Y.G. et al., 2014; Yang et al., 2017). Several studies have shown that DL-NBP can protect against cognitive impairment in vascular dementia (Liao et al., 2009; Jia et al., 2016; Qi et al., 2018). Whether DL-NBP can reduce or prevent the cognitive decline in AD is unclear. Excitingly, DL-NBP significantly improved cognitive function in rats injected with amyloid-β (Aβ) peptide as well as in a mouse AD model carrying mutations in the genes encoding amyloid precursor protein and presenilin 1 (Peng et al., 2009; Wang et al., 2016). These results suggest that DL-NBP may have a therapeutic efficacy for AD.

In the present study, we investigated the effects of DL-NBP on spatial learning and memory performance, and structural synaptic plasticity in SAMP8 mice. To explore the potential of DL-NBP in AD prevention and treatment, we also examined the DL-NBP-induced changes in synaptic protein levels and BDNF/TrkB signaling.

## Materials and Methods

### Drug Administration

**FIGURE 1:**
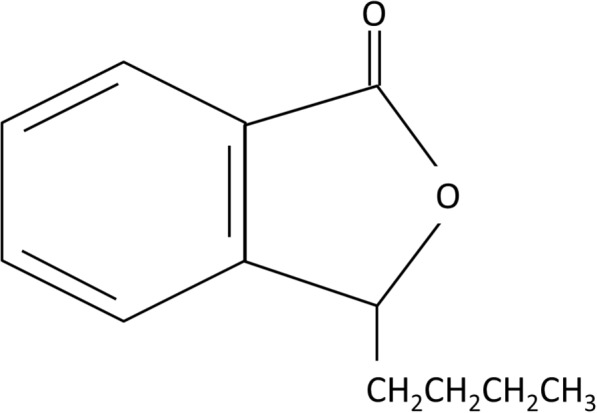
Chemical structure of DL-3-*n*-butylphthalide (DL-NBP).

DL-3-*n*-butylphthalide with a purity of 99.6% was provided by Shijiazhuang Pharma Group NBP Pharmaceutical Co., Ltd (Shijiazhuang, Hebei, China). The chemical structure of DL-NBP is shown in **Figure 1**. The compound was dissolved in vegetable oil at a working concentration of 20 or 40 mg/mL. The diluted DL-NBP was used for oral management at 2 mL/kg body weight. The drug was administered in two dosages, 40 mg/kg (low) and 80 mg/kg (high) once daily, in accordance with previous studies (Wang F. et al., 2014; Qi et al., 2018). The weight of each mouse was recorded weekly to determine the quantity of DL-NBP required for each dosage. Notably, the high dosage of DL-NBP was equal to that used for human clinical applications.

### Animal Treatment and Experimental Schedule

Male SAMR1 and SAMP8 mice were purchased from the Medical Department of Beijing University (Beijing, China). Animal experiments were performed under an animal study protocol approved by the Ethics Committee of Laboratory Animals at the First Hospital of Hebei Medical University. We strictly abided by the guidelines for the management and use of experimental animals in the present study. Compared with SAMR1 mice, SAMP8 mice show spatial learning and memory impairment starting from 4 months of age (Takeda, 1999). Therefore, we used 4-month-old male mice to study the effects of DL-NBP. The changes in behavior and synaptic protein levels were assessed at the age of 7 months, after 3-month treatment. Following 1 week of habituation to the cages, 4-month-old SAMP8 and SAMR1 mice were randomly divided each into three groups and orally administered DL-NBP or vegetable oil (2 mL/kg body weight) for 3 months. All animals were maintained with food and water *ad libitum*, and housed in cages at a constant temperature of 22 ± 2°C with a 12/12 h light/dark cycle.

**FIGURE 2:**
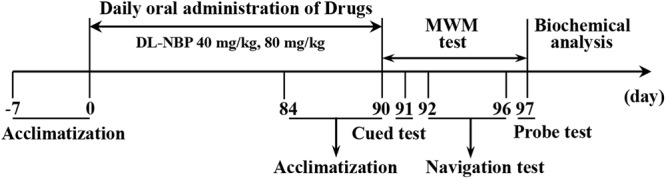
A schematic overview of the behavior and biochemical analysis in the study.

The designs of the behavioral and biochemical analyses are shown in **Figure 2**. After 1 week of acclimatization to the testing room, cognitive function was evaluated in the Morris water maze (MWM). A series of brain biochemical markers were studied 24 h after the MWM task.

### Behavioral Testing

To assess the spatial learning/memory ability of mice, MWM testing was carried out. The circular tank was 120 cm in diameter and filled with water (20–22°C). The water surface was divided into four virtual quadrants, and a platform (8 cm in diameter) was located in the middle of the target (“Southwest”) quadrant. On day 1, mice were trained to find a visible platform identical to the hidden platform used in the test (Vorhees and Williams, 2006). The curtains were drawn around the tank during this cued test to diminish the extra-maze cues in the room. During the following 5 days (orientation navigation test), the tank was surrounded by unique geometric shapes to support allocentric spatial navigation. Each mouse underwent four tests per day, with each test lasting <60 s and a 30-min intertrial interval, after which it was placed back in the cage under a heater to dry. To exclude egocentric learning strategies, the mice were placed in a different starting position in each of the four tests. Escape latencies to swim to the platform were recorded. Probe trials were performed 24 h after the orientation navigation test to determine memory retention. In these trials, the platform was removed, and the mice were placed in the tank at the side opposite to the quadrant where the platform had been located originally. Each mouse was allowed 60 s to explore, and the swimming speed, frequency of crossing the location of the target platform, and the time spent in each quadrant were recorded by the ANY-maze Behavior Analysis System (Stoelting, United States).

### Western Blotting

Hippocampi were extracted from mice rapidly and stored at -80°C. The tissues were cut into small fragments, and a moderate amount of cold tissue lysis buffer (3 mL of RIPA buffer with 30 μL of PMSF per gram of tissue) was added. The tissues were fully homogenized with a mortar grinder. The supernatants were collected after centrifugation for 10 min at 14,000 *g* at 4°C for further use. The protein concentrations were determined by a BCA kit (Solarbio, Beijing, China). Equal quantities of protein (30–60 μg/lane) were separated on an 8 or 10% SDS-PAGE gel and transferred onto PVDF membrane. The membranes were blocked in 5% skim milk for 2 h at 37°C, and then incubated at 4°C overnight with primary antibodies. The antibodies were purchased from Abcam (Cambridge, MA, United States) and included rabbit anti-PSD95 (1:5,000, ab76115), anti-SYN (1:10,000, ab32127), anti-BDNF(1:2,000, ab108319), anti-TrkB (1:1,000, ab18987), anti-cAMP-response-element-binding protein (CREB) (1:1,000, ab32515), and anti-phosphorylated CREB (pCREB) (1:5,000, ab32096). A primary antibody against β-actin was purchased from Affbiotech (Cincinnati, OH, United States). After three washes, the membranes were incubated with secondary antibody for 1 h. All bands were detected using an Odyssey CLX instrument (LI-COR, Lincoln, NE, United States). The gray level of each band was analyzed by the Image J software. The band intensities were normalized to that of β-actin.

### Quantitative Real-Time PCR

Total RNA was extracted using Trizol Reagent (Invitrogen) and treated with DNase I to remove genomic DNA. Quantitative real-time reverse-transcriptase PCR was performed following the manufacturer’s instructions for the Bio-Rad thermocycler and SYBR green kit (Invitrogen). Relative mRNA levels were normalized to that of β-actin and expressed as a percentage of the SAMR1 vehicle-treated samples. The following sequence-specific primers were used: PSD95 (5′-ACATTGGAAAGGGGTAAC-3′ and 5′-TAAAGATGGATGGGTCGT-3′), SYN (5′-AGTGGTGACTGGTGTGAC-3′ and 5′-GATTCCTTCCTGTGGGTA-3′), BDNF (5′-CTGGCTGACACTTTTGAG-3′ and 5′-TTATGGTTTTCTTCGTTG-3′), CREB (5′-AGTTGTTATGGCGTCCTC-3′ and 5′-TGTTTTGTTTTGGTTTTC-3′), β-actin (5′-GGCACCACACCTTCTAC-3′ and 5′-CTGGGTCATCTTTTCAC-3′).

### Statistical Analysis

All analyses were carried out using SPSS 21.0 (IBM software) and GraphPad Prism 5 (GraphPad software). Data are presented as means ± standard errors of the mean. Differences with *p* < 0.05 were considered statistically significant. Treatment (Vehicle vs. DL-NBP) and strain (SAMR1 vs. SAMP8) were used as independent factors in two-way ANOVA, followed by the Bonferroni *post hoc* correction. Studentized residuals were used to determine the abnormal value and Shapiro–Wilk’s test was used to test the normality of Student residuals. Escape latency in the MWM were evaluated using repeated-measures ANOVA, with days as the repeated factor.

## Results

### DL-NBP Alleviates the Cognitive Decline in SAMP8 Mice

Throughout the NBP treatment period, the health status of the mice was monitored closely. None of the mice showed behavioral or neurological abnormalities.

**FIGURE 3:**
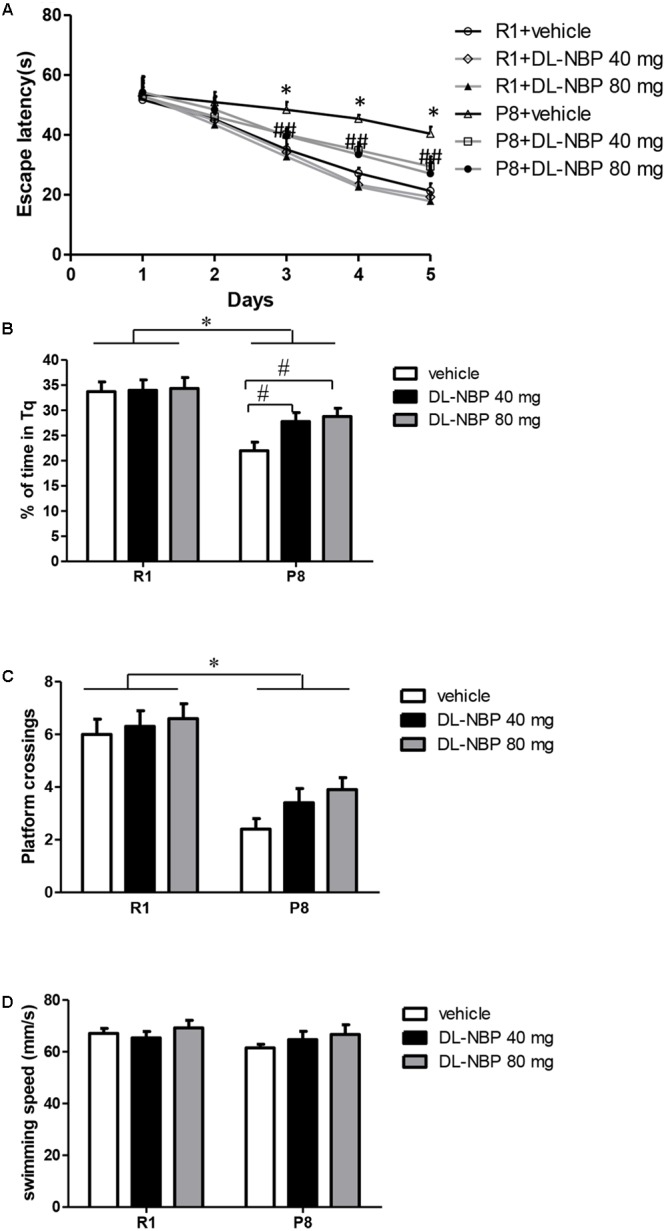
DL-3-*n*-butylphthalide (DL-NBP) improves spatial learning and memory in senescence-accelerated mouse prone 8 (SAMP8) mice in the Morris water maze. **(A)** Performance in orientation navigation tests evaluated by the average escape latencies of five consecutive days of four trials each. **(B,C)** Performance in the probe trial of each group evaluated by the percentage of target quadrant search time **(B)** and platform location crossings on day 6 **(C)**. **(D)** Swimming speed of each group in the MWM test. Statistical significance was determined using repeated-measures ANOVA with *post hoc* Bonferroni tests. Data are presented as the mean ± standard error of the mean (SEM). ^∗^*p* < 0.01 versus R1 group, ^#^*p* < 0.01, BL-NBP 40 mg versus P8 vehicle group, and ^##^*p* < 0.01, BL-NBP 80 mg versus P8 vehicle group. Tq, target quadrant; R1, SAMR1 mice; P8, SAMP8 mice (*n* = 10).

To study the effects of DL-NBP on cognitive function in SAMP8 mice, we exposed 4-month-old SAMP8 and SAMR1 mice to DL-NBP for 3 months, and then evaluated their behavior by the MWM test. Overall, no significant difference in swimming speed was observed between the groups during the entire MWM task [strain, *F* = 1.750, *p* = 0.218, treatment, *F* = 1.163, *p* = 0.335, interaction, *F* = 0.425, *p* = 0.579, **Figure 3D**]. To assess spatial learning and memory formation, orientation navigation tests were carried out for five consecutive days, and the escape latency was recorded. As shown in **Figure 3A**, the vehicle-treated SAMP8 mice showed a longer escape latency than did the SAMR1 mice on days 3 [*F* = 176.339, *p* < 0.001], 4 [*F* = 1090.152, *p* < 0.001], and 5 [*F* = 514.288, *p* < 0.001]. In SAMP8 mice, DL-NBP treatment shortened the escape latency relative to that in the vehicle-treated group on days 3 [*F* = 50.022, *p* < 0.001], 4 [*F* = 69.923, *p* < 0.001], and 5 [*F* = 46.628, *p* < 0.001]. In the probe trials on day 6, the percentage of target quadrant search time in the SAMP8 mice was markedly lower than in the SAMR1 mice [*F* = 12.179, *p* = 0.007, **Figure 3B**]. An obvious improvement of the SAMP8 mice was observed after DL-NBP treatment [*F* = 7.357, *p* = 0.005, **Figure 3B**]. The number of crossings over the target platform location in the SAMP8 mice was significantly lower than that in the SAMR1 mice [*F* = 45.451, *p* < 0.001, **Figure 3C**]. DL-NBP treatment did not significantly increase the number of target crossings in the SAMP8 mice, although the group did show a trend-level improvement [*F* = 2.203, *p* = 0.139, **Figure 3C**]. No marked differences related to DL-NBP administration were seen in the escape latency, percentage of target quadrant search time, and the number of platform crossings of the SAMR1 mice (*p* > 0.05). No difference between the two DL-NBP dosages in the navigation tests or probe trials were observed (*p* > 0.05). Taken together, our data show that DL-NBP significantly reduced the spatial learning and memory deficits of SAMP8 mice.

### DL-NBP Improves Structural Synaptic Plasticity in the Hippocampus of SAMP8 Mice

**FIGURE 4:**
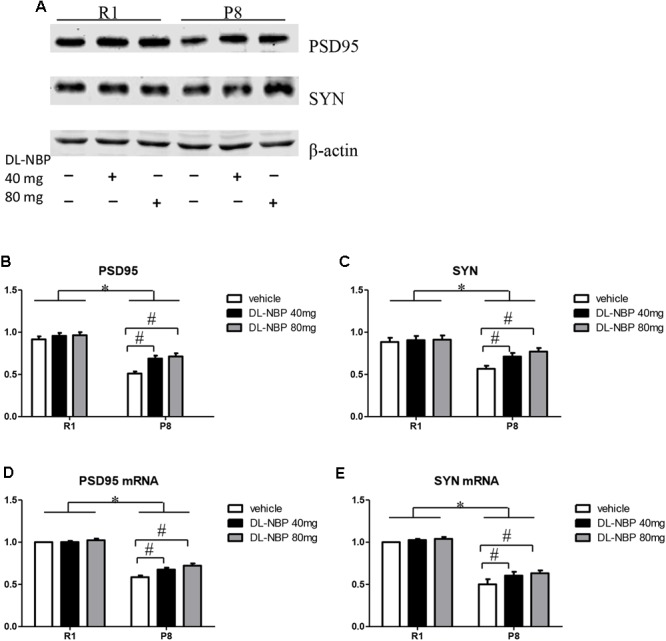
Effects of DL-3-*n*-butylphthalide (DL-NBP) management on structural synaptic plasticity in the hippocampus of SAMP8 mice. **(A–C)** Representative western blot images of synaptophysin (SYN) and postsynaptic density protein 95 (PSD95). **(D,E)** SYN and PSD95 mRNA levels in the SAMP8 hippocampus. Values were normalized to β-actin and the mRNAs were expressed as a percentage of the SAMR1 vehicle-treated samples. Data are presented as the mean ± standard error of the mean (SEM). ^∗^*p* < 0.01 versus R1 group, ^#^*p* < 0.01 versus P8 vehicle group. R1, SAMR1 mice; P8, SAMP8 mice (*n* = 5).

Because DL-NBP reduced the cognitive decline in SAMP8 mice as assessed by the MWM test, we next evaluated the levels of hippocampal synaptic proteins forming the structural basis of synaptic plasticity in learning and memory. As shown in **Figure 4**, western blot analysis showed a pronounced decrease in the levels of PSD95 and SYN in the SAMP8 mice compared to those in the SAMR1 group [*F* = 252.405, *p* < 0.001, **Figure 4B**; *F* = 189.742, *p* < 0.001, **Figure 4C**]. Remarkably, the levels of both proteins were significantly increased by DL-NBP treatment in SAMP8 mice [*F* = 107.951, *p* < 0.001, **Figure 4B**; *F* = 81.031, *p* < 0.001, **Figure 4C**], whereas the SAMR1 mice showed no significant changes (*p* > 0.05). Consistent with the western blotting data, real-time PCR analysis showed that the levels of PSD95 and SYN mRNAs in the SAMP8 mice were lower than those in the SAMR1 mice [*F* = 353.950, *p* < 0.001, **Figure 4D**; *F* = 66.966, *p* = 0.001, **Figure 4E**]. DL-NBP administration caused an increase in the PSD95 and SYN mRNA levels in the SAMP8 mice [*F* = 23.661, *p* < 0.001, **Figure 4D**; *F* = 13.305, *p* = 0.003, **Figure 4E**], whereas the SAMR1 mice showed no significant changes (*p* > 0.05). Taken together, these results demonstrate that DL-NBP treatment substantially increased PSD95 and SYN expression at the protein and mRNA levels in SAMP8 mice, suggesting that DL-NBP likely alleviates the decline in MWM performance in these mice by increasing hippocampal synaptic protein expression.

### DL-NBP Enhances BDNF/TrkB Signaling in the Hippocampus of SAMP8 Mice

**FIGURE 5:**
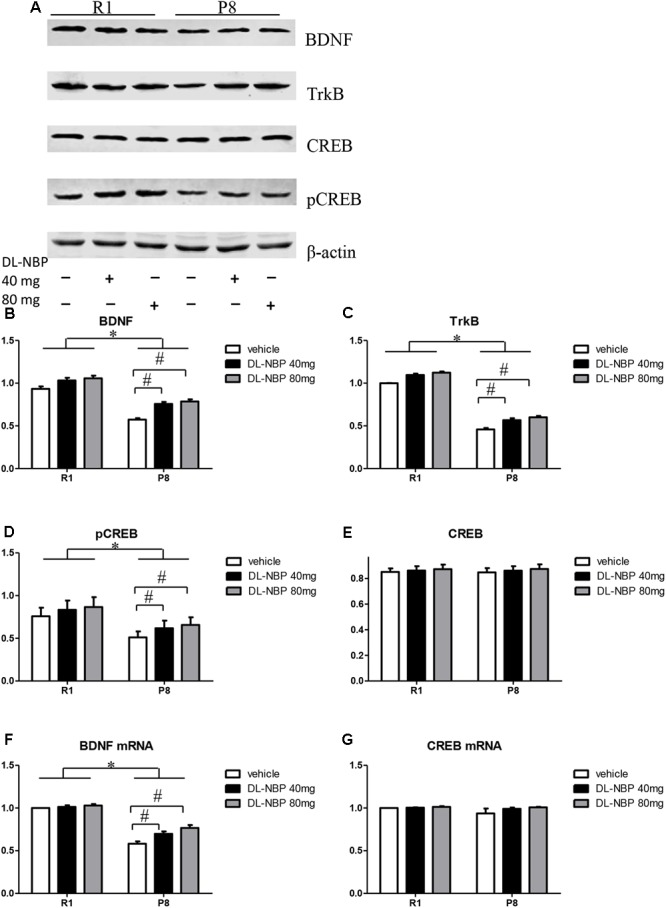
Effects of DL-3-*n*-butylphthalide (DL-NBP) treatment on brain-derived neurotrophic factor (BDNF)/tropomyosin receptor kinase B (TrkB) signaling in the hippocampus of SAMP8 mice. **(A–E)** Representative western blot images of BDNF, TrkB, cAMP-response-element-binding protein (CREB), and phosphorylated CREB (pCREB). **(F,G)** BDNF and CREB mRNA levels evaluated by real-time PCR. Values were normalized to β-actin and the mRNAs were expressed as a percentage of the SAMR1 vehicle-treated samples. Data are presented as the mean ± standard error of the mean (SEM). ^∗^*p* < 0.01 versus R1 group, ^#^*p* < 0.01 versus P8 vehicle group. R1, SAMR1 mice; P8, SAMP8 mice (*n* = 5).

Studies have shown that BDNF can effectively regulate synaptic plasticity, learning, and memory. The distribution and expression of synaptic proteins were regulated via BDNF binding to its receptor TrkB, whereas the neuroprotective effects of BDNF were dependent on CREB-mediated transcription. We investigated the effects of DL-NBP treatment on BDNF/TrkB signaling in the hippocampus of SAMP8 mice. Western blot analysis showed a marked decrease in the levels of BNDF and TrkB in the SAMP8 mice compared with those in the SAMR1 group [*F* = 681.100, *p* < 0.001, **Figures 5A,B**; *F* = 1564.989, *p* < 0.001, **Figures 5A,C**]. Both protein levels were significantly increased by DL-NBP treatment in the SAMP8 mice [*F* = 850.925, *p* < 0.001, **Figures 5A,B**; *F* = 59.126, *p* < 0.001, **Figures 5A,C**], whereas the SAMR1 mice showed no significant changes (*p* > 0.05). Real-time PCR analysis showed that the levels of BDNF mRNA in the SAMP8 mice were lower than those in the SAMR1 group [*F* = 178.015, *p* < 0.001, **Figure 5F**]. The levels of BDNF mRNA were significantly increased by DL-NBP treatment in the SAMP8 [*F* = 26.343, *p* < 0.001, **Figure 5F**]. DL-NBP treatment did not change the total levels of CREB as assessed by western blotting or real-time PCR (*p* > 0.05, **Figures 5A,E,G**). The levels of pCREB in the SAMP8 mice were markedly lower compared with those in the SAMR1 group [*F* = 39.972, *p* < 0.001, **Figures 5A,D**] and were significantly increased by DL-NBP treatment in the SAMP8 mice [*F* = 39.972, *p* < 0.001, **Figures 5A,D**], whereas the SAMR1 mice showed no significant changes (*p* > 0.05). These results suggest that DL-NBP greatly enhanced BDNF/TrkB signaling in the hippocampus of SAMP8 mice, which is a potential mechanism of the DL-NBP-induced protection of synaptic plasticity.

## Discussion

Multiple challenges hinder the development of new medications protecting against the AD-related cognitive decline, including extended process length, high costs, and low success probability. Therefore, repurposing older drugs to treat AD may represent a valid alternative. DL-NBP has been widely used in the treatment of ischemic cerebral apoplexy, in which it improves the brain microcirculation and reduces mitochondrial and oxidative damage. Our results indicate that DL-NBP markedly alleviates the cognitive and memory dysfunction in SAMP8 mice, an animal model of AD.

SAMP8 mice are characterized by early-onset, rapidly advancing senescence and display pronounced age-related deprivations in learning and memory ability. The SAMP8 is an excellent model in which to study the behavioral consequences of, and test therapeutic interventions for, AD. The hippocampus is one of the most age-sensitive areas of the brain. The aging process severely weakens hippocampal plasticity, which leads to cognitive impairment (Driscoll and Sutherland, 2005; Takeda and Tamano, 2014; Chang et al., 2016). In MWM tests, SAMP8 mice showed impaired spatial learning and memory formation starting from the age of 4 months, as compared with the control SAMR1 mice (Takeda, 1999). DL-NBP administration for 3 months alleviated these deficits in SAMP8 mice. Memory retention was also decreased in SAMP8 mice, manifesting as a reduced percentage of target quadrant search time and a lower number of platform location crossings in the probe trial. However, DL-NBP treatment did not increase the number of platform crossings in SAMP8 mice, possibly because of their low baseline frequency (Vorhees and Williams, 2006). In contrast, DL-NBP treatment markedly increased the percentage of target quadrant search time in SAMP8 mice. Taken together, our results show that DL-NBP greatly improves learning and memory functions in SAMP8 mice.

Synaptic plasticity is one of the most important features of the nervous system and serves as the foundation of learning and memory consolidation (Abbott and Nelson, 2000; Citri and Malenka, 2008; Sehgal et al., 2013). SYN and PSD-95 are markers for the pre- and postsynaptic terminals, respectively, which represent the structural bases of plasticity underlying learning and memory. SYN expression reflects the density and distribution of synapses and is closely related to their formation and reconstruction (Kwon and Chapman, 2011; Xia et al., 2017). SYN may not only affect the synaptic structure directly, but may also affect synaptic plasticity by regulating neurotransmitter release (Sudhof and Rothman, 2009). The PSD is the structural basis of postsynaptic plasticity. The PSD may regulate the structure of synaptic connections and transduce membrane receptor signals by interacting with a variety of proteins (Migaud et al., 1998; Lin et al., 2016). The PSD is also an important site for information transmission and memory formation. The reduction of PSD95 levels in the hippocampus can lead to impairment of learning and memory function. It is noteworthy that the decline of synaptic function in AD occurs before the deposition of Aβ plaques (Mucke et al., 2000). Oxidative damage has also been observed at the early stages of AD pathology. Extensive oxidative damage is present in brains with mild cognitive impairment and is particularly prominent in AD brain synapses (Lovell and Markesbery, 2001; Ansari and Scheff, 2010). Studies have revealed that SAMP8 mice show increased oxidative stress as early as at 2 months (Yasui et al., 2003). DL-NBP has been reported to reduce ischemic cerebral injury by alleviating oxidative stress and mitochondrial damage (Li et al., 2009). DL-NBP pretreatment not only preserved the mitochondrial membrane potential, but also reduced ROS generation *in vitro* (Xiong et al., 2012). Our results show that the levels of synaptic proteins were significantly reduced in the SAMP8 mice relative to those in SAMR1 mice. Excitingly, after 3 months of DL-NBP administration, the SAMP8 mice showed significantly increased SYN and PSD95 levels. These results are consistent with those of the MWM tests and suggest that DL-NBP may protect synaptic function in the hippocampus. Whether the protection is mediated by the antioxidant properties of the compound needs further study.

Multiple studies have demonstrated that BDNF expression is dynamically regulated by neuronal activity and the Ca^2+^ concentration. The activity-dependent secretion pattern of BDNF/TrkB signaling may modulate synapse formation and plasticity, and therefore the acquisition and consolidation of memory. Consistent with this hypothesis, BDNF and TrkB were widely located in the axon terminals (presynaptic sites) and dendrites (postsynaptic sites) of hippocampal neurons. The distribution and expression of synaptic proteins, such as SYN and PSD95, may be modulated by BDNF/TrkB signaling (Jovanovic et al., 2000; Chen et al., 2017). It is well known that pCREB regulates the transcription of downstream genes including *BDNF* (Reichardt, 2006; Lopez de Armentia et al., 2007). CREB is a transcription factor crucial for regulating synaptic plasticity and cognitive function (Belgacem and Borodinsky, 2017; Gite et al., 2018). The expression of CREB in the hippocampus is decreased in the AD brain (Satoh et al., 2009). Increasing CREB levels can reduce the cognitive impairment in an AD mouse model (Yiu et al., 2011). Several studies have shown that CREB plays a major role in regulating ROS toxicity and antioxidant gene expression (Bedogni et al., 2003; Kronke et al., 2003; Zou and Crews, 2006). Recent work also suggests that BDNF preserves cognitive performance via the attenuation of oxidative stress, and the neuroprotective effects of BDNF are dependent on CREB-mediated transcription (Lee et al., 2009).

In the present study, the protein or mRNA levels of BDNF and TrkB were reduced in the hippocampus of SAMP8 mice relative to those in age-matched SAMR1 mice. This decrease was relieved by 3-month DL-NBP treatment. In addition, the levels of pCREB were also markedly decreased in the SAMP8 mice compared with those in the SAMR1 group, and significantly increased following DL-NBP treatment. Collectively, these data strongly suggest that DL-NBP may protect synaptic function by enhancing BDNF/TrkB signaling and thus reducing oxidative stress. Notably, DL-NBP treatment did not change the total levels of CREB at either the protein level or transcript level. One possible explanation for this phenomenon is that the biological activity of CREB is regulated by various protein kinases, such as protein kinases A and C, mitogen-activated protein kinase, Ca^2+^/calmodulin-dependent kinase, and casein kinase II, which play different roles in CREB expression (Michel et al., 2008). The influence of DL-NBP on the activities of these upstream CREB regulators needs to be further investigated.

## Conclusion

Long-term DL-NBP treatment reduced the cognitive decline in SAMP8 mice by regulating structural synaptic plasticity, likely via enhancement of BDNF/TrkB signaling. Clarification of these effects will help illuminate the complex mechanisms induced by DL-NBP and may serve as the foundation for the development of effective drugs capable of improving cognitive function in age-related dementia. Because the mild cognitive impairment in 4-month-old SAMP8 mice used in the present study is equivalent to the early stages of AD in human patients, our findings are likely to be informative for research into potential AD therapies. Further studies of the effects of DL-NBP on AD pathology are required to better understand the potential of DL-NBP in protecting human patients with AD from cognitive decline.

## Author Contributions

CL designed the experiments and drafted the manuscript. CL, JL, and XZ performed the experiments. QM, YG, and BH assisted in the design of the study, statistical analysis, and manuscript writing. MW revised the work and agreed to be accountable for all aspects of the work.

## Conflict of Interest Statement

The authors declare that the research was conducted in the absence of any commercial or financial relationships that could be construed as a potential conflict of interest.
